# An inter-residue network model to identify mutational-constrained regions on the Ebola coat glycoprotein

**DOI:** 10.1038/srep45886

**Published:** 2017-04-11

**Authors:** Devin S. Quinlan, Rahul Raman, Kannan Tharakaraman, Vidya Subramanian, Gabriella del Hierro, Ram Sasisekharan

**Affiliations:** 1 Department of Biological Engineering, Massachusetts Institute of Technology, Cambridge, MA, USA; 2 Koch Institute for Integrative Cancer Research, Massachusetts Institute of Technology, Cambridge, MA, USA; 3 Department of Chemical Engineering, Massachusetts Institute of Technology, Cambridge, MA, USA

## Abstract

Recently, progress has been made in the development of vaccines and monoclonal antibody cocktails that target the Ebola coat glycoprotein (GP). Based on the mutation rates for Ebola virus given its natural sequence evolution, these treatment strategies are likely to impose additional selection pressure to drive acquisition of mutations in GP that escape neutralization. Given the high degree of sequence conservation among GP of Ebola viruses, it would be challenging to determine the propensity of acquiring mutations in response to vaccine or treatment with one or a cocktail of monoclonal antibodies. In this study, we analyzed the mutability of each residue using an approach that captures the structural constraints on mutability based on the extent of its inter-residue interaction network within the three-dimensional structure of the trimeric GP. This analysis showed two distinct clusters of highly networked residues along the GP_1_-GP_2_ interface, part of which overlapped with epitope surfaces of known neutralizing antibodies. This network approach also permitted us to identify additional residues in the network of the known hotspot residues of different anti-Ebola antibodies that would impact antibody-epitope interactions.

Ebola virus (EBOV) is a negative-strand RNA virus that originated in equatorial Africa, and together with the Marburg virus, comprises the viral family *Filoviridae*. Infection with these viruses causes a severe hemorrhagic fever in humans, with mortality rates as high as 90%. Since the first outbreaks of Sudan ebolavirus (SUDV) and Zaire ebolavirus (EBOV) in 1976, additional strains have been identified from subsequent incidents and outbreaks: Tai Forest (TAFV), Bundibugyo (BDBV), and Reston (RESTV), though SUDV and EBOV remain the most prevalent^1^. Furthermore, the frequency with which outbreaks of both of these strains occur has increased since their discovery, with major outbreaks occurring from 1994–1997, 2000–2004, and 2007–2009. This trend has culminated in the most recent 2014 EBOV outbreak, in which, as of April 2016, over 28,000 cases (15,000 laboratory-confirmed) and 11,000 deaths have been reported – an order of magnitude higher than all previous outbreaks combined^2^.

The Ebola virus surface glycoprotein (GP) is a homotrimer that plays a key role in viral entry, and it also presents the predominant surface epitopes for neutralization by therapeutic antibodies, vaccines and host immune response. The mature virion surface GP has two subunits, GP_1_ and GP_2_. GP_1_ contains both the glycan cap and mucin domains, which are heavily glycosylated and important for receptor binding, while GP_2_ is involved in mediating fusion and viral entry^3^,^4^. The GP gene is affected by RNA editing, and thus encodes for the production of three separate protein species, the 676-residue structural, trimeric GP that forms the viral envelope (GP), a 364-residue secreted, dimeric form (sGP), and a 298-residue small secreted form (ssGP)^5^,^6^. Additionally, trimeric GP also exists in several states throughout its life cycle as the result of enzymatic cleavage—first by furin in the Golgi to create the GP_1_ and GP_2_ subunits, and then by cathepsins which remove glycan cap and mucin domain in the endosome of the targeted cell to allow for viral fusion and entry^7^. The multiple forms of GP resulting from its production and post-processing in the host cell makes it a difficult target for therapeutic- or vaccine-based interventions. However there has been significant progress in development of multiple therapeutic antibodies targeting different regions of GP, which have shown efficacy in animal models when given as a cocktail. Beginning with the KZ52 antibody, which was isolated from a human survivor in 1995, monoclonal antibody cocktails have been developed and reformulated, including the MB-003 cocktail, which contains the 6D8, 13C6 and 13F6 mAbs, the ZMAb cocktail, which contains the 1H3, 2G4 and 4G7 mAbs, and the ZMapp cockail, which contains of the 2G4, 4G7 and 13C6 antibodies^8^,^9^,^10^. Recently, new antibodies have been identified following the 2014–2015 outbreak that could improve upon the ZMapp cocktail^11^,^12^,^13^,^14^,^15^,^16^,^17^. These cocktails, in addition to the progress on an Ebola vaccine, have been very promising in the context of preventing future outbreaks^18^.

The evolutionary pressure of EBOV has not resulted in significant antigenic drift in terms of sequence variation of GP given the localized outbreaks and long time frame between large outbreaks. As a result, there is a high degree of sequence conservation across reported EBOV GP (Fig. 1A). However, the reported nucleotide substitution rate is very high, at 1.25 × 10^−3^ substitutions per site per year^19^, a value which is on par with that of other viruses, such as Dengue and influenza (Supplementary Table S1). The high sequence conservation among GP, taken together with selection pressure resulting from therapeutic antibody development, necessitate a different approach to understand propensity of GP residues to mutate so as to escape neutralization.

Towards this goal, we employed an approach we had developed previously^20^ to map in a two-dimensional space the inter-residue interaction network of each GP residue by analyzing all the available X-ray crystal structures of the processed trimeric GP presented as GP_1_ and GP_2_ on the surface of the mature virion. This approach permitted us to capture the structural constraints on the mutability of solvent-exposed surface residues on the GP trimer imposed by the extent of their inter-residue interaction network. Using this approach, we identified two distinct clusters along the interface of GP_1_-GP_2_ on the trimeric GP surface that are comprised of highly networked residues that are constrained to mutate. These regions may present new epitope surfaces for potent neutralization by one or more therapeutic antibodies. The network map of GP surface residues also presented new insights on the epitope regions that are targeted by known anti-GP antibodies such as 2G4, 4G7, KZ52, etc. Specifically, we identified additional residues in the network of known hotspots on epitopes that could significantly impact antibody binding. Taken together, our approach provides a rational way to structurally define constraints on mutability of viral surface proteins and also provides new insights into epitope regions on these proteins from a network perspective.

## Results

### Structural constraints on mutability of residues on surface of EBOV GP

To define constraints on mutability of residues in EBOV GP, we computed the inter-residue interaction network from the available X-ray crystal structures. Briefly, this residue interaction network is a method for numerically describing the interconnectedness of various residues found within a protein crystal structure. Network score attempts to capture not only the directly bonded residue pairs, but those pairs of residues whose connection is mediated by a common bonded residue or series of residues. In this way, the network score describes more than the bond strength of a residue and its neighbors but the full bond environment of a residue. Taking the sum total of the network scores between a given residue and all of those residues within its environment produces a score which can be thought of as a measure of total connectedness for the given residue. These network scores are then normalized to scale of 0–1, where 1 represents maximum connectivity in terms of inter-residue interactions. A low score indicates that a residue is not as connected and hence is structurally less constrained to mutate.

**Figure 1:**
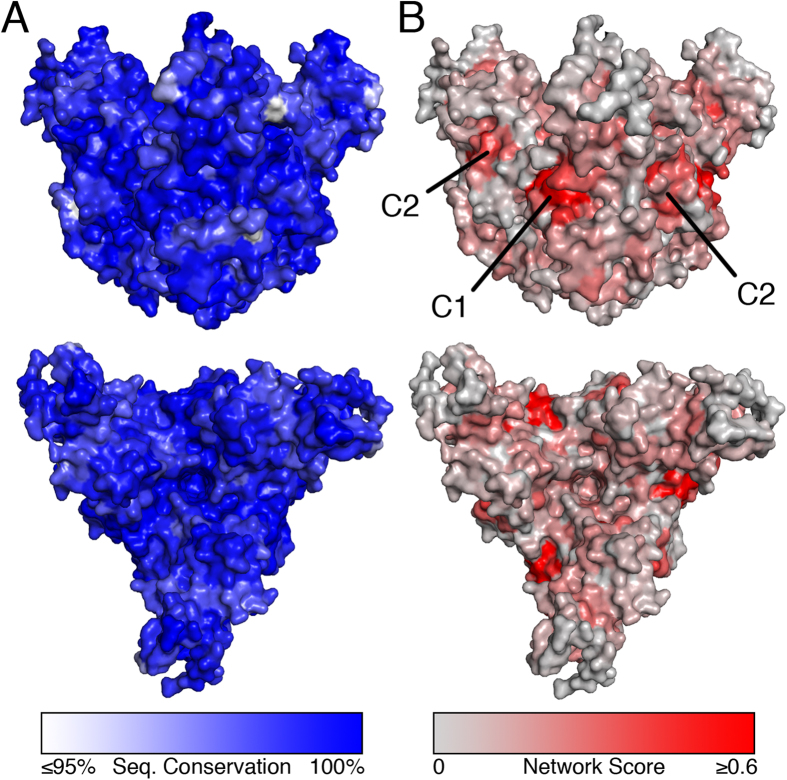
Comparison between sequence conservation and network score analysis of GP surface. (**A**) Sequence conservation is mapped to the GP surface using a gradient ranging from ≤95% conserved (white) to 100% conserved (blue). (**B**) Normalized network scores are mapped to the GP surface using a gradient ranging form 0 (gray) to ≥0.6 (red). Gradient cutoffs were selected to highlight relevant surface trends and reduce skewing from outliers.

The network scores were mapped on the protein surface using a threshold solvent accessible surface area (Fig. 1B), and are contrasted to a similar figure created by instead using the protein sequence conservation percentage of EBOV GP (Fig. 1A). The network map permitted identification of two distinct clusters of residues with high network score on opposing faces of each monomer in the GP trimer, denoted as C1 and C2. C1 includes residues W104, E106, R134, R136, H516 and E545, while C2 includes residues R85, K155, E156, L529, Y534 and F535. Each of these lies along the GP_1_-GP_2_ interface, and is below the cathepsin-cleavage site (which cleaves just below the glycan cap). These clusters comprise of residues that are highly constrained to mutate either through natural evolution or through selection pressure by therapeutic antibodies designed to target these regions. Though there is limited information on the functional importance of these clusters, both C1 and C2 contain residues that have been described as being part of the viral fusion loop for EBOV GP (Supplementary Fig. S1)^3^. Our analysis showed that the GP_1_-GP_2_ interface region, which forms the base of the GP trimer, has more residues that are structurally constrained compared to the regions on GP_1_ that form the glycan cap.

**Figure 2:**
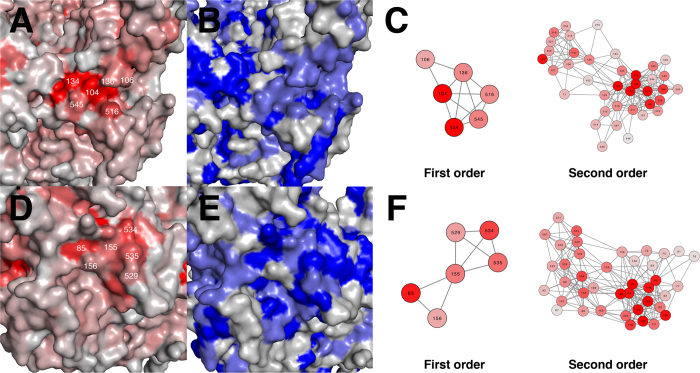
Networked clusters on GP surface. Two highly networked clusters are shown on the GP surface. GP is colored either by network score (**A**,**D**) or by sequence conservation percentage across filovirus strains (**B**,**E**). Residue connectivity diagrams are also shown for each cluster for both the first and second order network, and colored according to network score. The first cluster (C1) contains residues 104, 106, 134, 136, 516 and 545 (**A**–**C**). The second cluster (C2) contains residues 85, 155, 156, 529, 534 and 535 (**D**–**F**). Network scores are colored from 0 (gray) to ≥ 0.6 (red). Sequence conservation is colored from ≤ 70% (gray) to 100% (blue).

To understand the relationship between network score and sequence conservation with regard to structurally constrained residues, we investigated the sequence diversity of the various coat glycoproteins across the filovirus family, which contains the Zaire, Sudan, Tai Forest, Reston and Bundibugyo strains of Ebola virus, as well as Marburg virus. We observed that the degree of conservation correlated with the network score of the residue (Supplementary Fig. S3). The two clusters with highly networked residues also overlapped with the highly conserved regions in GP (Fig. 2). In the case of C2, many of the residues were as high as 100% conserved across all filoviruses.

### Analysis of antibody-epitope interactions in context of network-mapped GP surface

**Figure 3:**
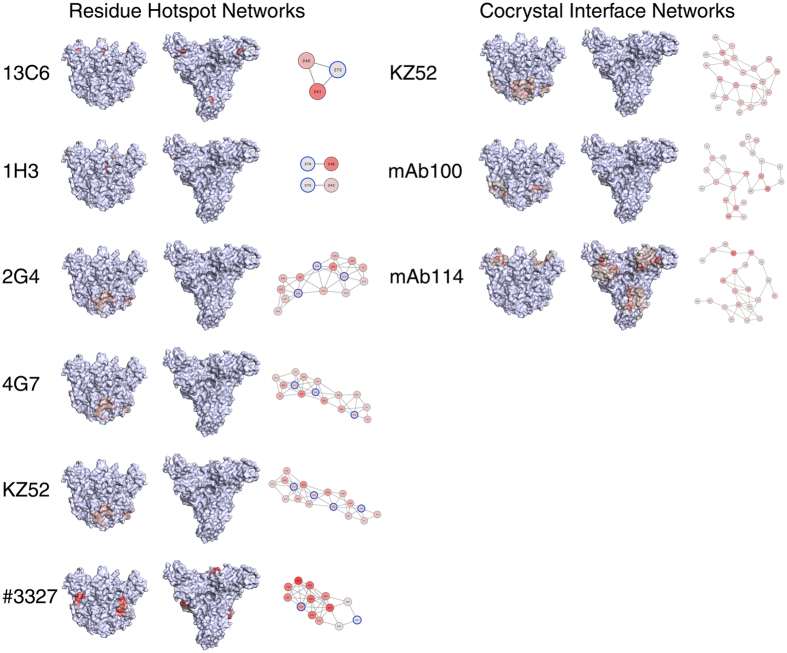
Antibody binding networks for anti-Ebola antibodies. 13C6, 1H3, 2G4, 4G7, KZ52, #3327, mAb100 and mAb114 epitope networks are mapped onto the surface of EBOV GP, which is shown from both a side view, top view, and network view (from left to right). Antibody epitopes are either represented as secondary networks derived from residue hotspot information (first column) or as interface networks derived from publically available crystal structures (second column). KZ52 is listed twice, as both types of data exist for this antibody. A blue circle surrounding a node indicates a hotspot residue that the second-order network graph was generated from. The residues within each epitope are colored by their network score, from 0 (gray) to ≤0.6 (red).

Having identified the regions on the surface of EBOV GP that are highly constrained, we next sought to investigate the relationship between these regions and the currently existing anti-GP antibodies. To date, there have been a variety of anti-GP antibodies identified, but the specific epitope information has only been identified in a smaller subset. We chose to look at antibodies for which specific residue information had been described, either through residue mutation and binding experiments, or through x-ray crystallography to look at GP-antibody binding. While additional studies have used electron micrography techniques to determine the general region of binding for a variety of anti-GP antibodies, we chose to exclude them from our analysis, as the residue information was not specific enough to enable reliable analysis. Thus, we investigated the network properties for the epitopes of the 13C6, 1H3, 2G4, 4G7, KZ52, #3327, mAb100 and mAb110 antibodies (Fig. 3, Supplementary Table S2). In the case of 13C6, 1H3, 2G4, 4G7, KZ52 and #3327, specific residues had been identified that directly impacted binding specificity (often termed ‘hotspots’). We used these residues, as well as the residues that were networked to those residues to analyze the footprint of a given antibody on the GP surface. In the case of mAb110 and mAb114, antibody cocrystal structures were available, so we analyzed the network of residues in GP that are specifically on the interface of the epitope-paratope interaction. We found that, across the antibodies, those that bound to the glycan cap (1H3, 13C6, mAb114) all had lower median network scores and reduced interconnectivity compared to antibodies that target epitopes along the GP_1_-GP_2_ interface, such as KZ52. Additionally, we saw that the antibody #3327 bound a region near cluster C2. While its hotspot glycine residue at position 528 makes no side chain interactions, incorporating the adjacent residues 527 and 529 into the analysis uncovered a secondary network that was highly interconnected and had the highest median network score, which is in agreement with our observation that it appeared to bind C2. 2G4, 4G7 and KZ52 antibodies were all observed to have highly similar network properties and median scores, which corresponds to the fact that these antibodies overlap and share several of the same hotspot residues. Interestingly, although mAb100 binds a region near the GP_1_-GP_2_ interface, the residues at the binding interface are relatively poorly networked, worse even than mAb114, which binds the glycan cap region.

### Network analyses of epitope hotspot residues

**Figure 4:**
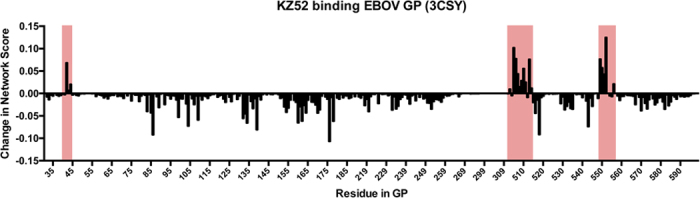
The difference in residue network scores between unbound GP and GP bound to KZ52. Each bar represents a single residue in EBOV GP. Pink regions highlight the residues at the interface of the GP-KZ52 interaction (PDB: 3CSY).

We sought to understand the effects of the known hotspots and the residues in their interaction network on antibody binding. Using the published cocrystal structure containing EBOV GP bound to Fab KZ52 (PDB: 3CSY), we computed the network scores for all residues, with and without including the binding of KZ52 (Fig. 4). We observed that within the epitope-paratope interface region, there were a number of residues on GP that experienced a large increase in network score when KZ52 was included in the analysis. This means that these residues were much more highly networked in the context of the bound protein complex. Interestingly, while many of these residues corresponded with known KZ52 hotspot residues, there were other residues observed that had high network scores in this region that were not observed to be hotspot residues in Davidson *et al*.^21^. To understand this discrepancy further, we generated a series of GP point mutants in the residue positions that are highly networked and screened these mutants for binding to a small panel of anti-GP antibodies that included KZ52, 13C6, 2G4 and 4G7. Briefly, we expressed a recombinant form of GP lacking the transmembrane and mucin domains (GPΔTMΔmuc), using a his-tag on the C-terminal end for purification. Site-directed mutagenesis was then used to create single point mutants for each site, in order to disrupt the binding network of an antibody-GP interaction. For the wild type GPΔTMΔmuc as well as for the mutants, we were able to obtain purified, trimeric protein (Supplementary Fig. S4). Finally, we measured the binding of the point mutants to each of the four anti-GP antibodies (Table 1).

We observed that for the each of the mutants tested, few of the mutants experienced a decrease in 13C6 binding greater than 3-fold compared to wild type. We used 13C6 as a control, since it binds an epitope on the GP surface away from the mutated regions. We observed that in addition to the known residue hotspots, 511, 550, 552, 553, and 556, the L547A and N506A mutations had drastic effects on the binding of these antibodies without drastically affecting 13C6 affinity. The L547A mutation primarily affected the binding of 4G7 (>100-fold reduction in affinity) while also having noticeable impacts (>10-fold) on KZ52 and 2G4 binding. Conversely, the N506A mutation primarily affected KZ52 and 2G4 binding (>100-fold) while only slightly affecting 4G7 binding (>3-fold).

## Discussion

The outbreak of EBOV is unique (when compared to other pathogens) in that it is highly localized, the mortality rate is very high, and there are no approved therapeutic or vaccine strategies. As a consequence, the sequence evolution of GP is slower owing to limited evolutionary and host selection pressure – although the mutation frequency is similar when compared to other pathogens, such as influenza A virus. Therefore there is a need to look beyond sequence evolution to understand constraints on mutability of amino acids in GP.

We have presented in this study a new approach to define constraints on amino acid mutability of GP based on their inter-residue interactions in three-dimensional space, which are mapped into a two-dimensional network. Our approach permits overcoming the limitation of understanding amino acid changes based on sequence evolution of EBOV. The strength of our approach is highlighted by our ability to identify *a priori* two distinct clusters C1 and C2 that comprised of residues that are highly constrained to mutate and hence present new targets for neutralization by therapeutic antibodies. In fact, our findings were independently validated by studies that identified antibodies that targeted novel neutralizing epitope regions that significantly overlapped with the C2 cluster^11^,^15^.

In addition to identifying highly networked clusters, our approach also provided new insights into the known epitope regions of existing antibodies. The antibodies that bound the glycan cap region of GP (1H3, 13C6 and mAb114) had low network scores and that the residues within the networks were not highly interconnected. Given our observations relating residue network score to filovirus sequence conservation (Supplementary Fig. S3), it is possible that these glycan cap-binding antibodies are more prone to antigenic escape. A similar problem exists for mAb110, which bound a region near the GP_1_-GP_2_ interface, but primarily interfaces with residues that are relatively poorly networked in the GP structure. These antibodies are contrasted to the constrained binding networks of the KZ52, 2G4, 4G7 and #3327 antibodies, the latter of which seems to engage with the highly constrained C2 cluster. Finally we have also expanded the hotspot regions based on the network analysis to include residues in addition to those identified by Davidson *et al*. Specifically the N506 residue appears to be a hotspot for the binding of KZ52 and 2G4, and the L547 residue appears to be a hotspot for 4G7. Compared to the method used in Davidson *et al*., we use a soluble expression of GPΔTMΔmuc, a construct missing the transmembrane and mucin domains, which could have affected antibody binding. Additionally, in Davidson *et al*., the authors used a flow cytometry approach and did not list data for the additional residues we identified. Given the reduced expression of some of these mutants, it is possible that they were discarded from their analysis.

Taken together, our analyses here have demonstrated that an inter-residue network approach is a useful methodology to gain insights about viral antigens from x-ray crystal structure data, without needing extensive evolutionary history or prior knowledge of antibody epitopes.

## Methods

### GP Expression and Purification

A pcDNA 3.3 expression vector containing a sequence encoding for GPΔTMΔmuc (missing transmembrane and mucin domain) was transiently transfected into HEK 293 F cells. After 6 days, supernatant was harvested and purified using a 1 mL HisTrap HP column on an AKTA FPLC (GE Healthcare). Fractions were collected and analyzed using Native PAGE. Fractions containing trimeric GP species were combined and buffer exchanged into PBS using Amicon Ultra Centrifugation Filters (Millipore). Protein concentration was determined using a BCA assay (Pierce) and GP was assessed again using Native PAGE to confirm purity.

### Antibody Expression and Purification

Similarly to the method for expressing GP, pcDNA 3.3 expression vectors encoding for anti-GP mAbs c13C6, c2G4 or c4G7 were transiently transfected into HEK 293 F cells. After 6 days, supernatant was harvested and purified using a 1 mL HiTrap Protein A column using an AKTA FPLC (GE Healthcare). Fractions containing antibody were collected and buffer exchanged into PBS. Protein concentration was determined using BCA assay.

### Site-directed mutagenesis

GPΔTMΔmuc point mutants were created using site-directed mutagenesis. Mutagenesis primers were designed corresponding to the mutant sequence, and a PCR amplification reaction was carried out using a QuikChange Mutagenesis Kit (Agilent). PCR reactions were then digested using Dpn1 for 3 hours, transformed into One Shot TOP10 Chemically Competent cells (Thermo) and then plated onto LB agar plates containing ampicillin. Plasmid DNA was generated by growing colonies in LB broth containing ampicillin overnight and then by purifying using plasmid DNA preparation kits (Invitrogen). Positive colonies were confirmed using Sanger sequencing (Genewiz).

### Enzyme-Linked Immunosorbent Assays (ELISA)

WT GPΔTMΔmuc or mutants were plated onto clear 96-well Maxisorp plates (Nunc) at a concentration of 1 μg/mL and left at 4 °C overnight. Plates were then washed with PBST (PBS with 0.05% tween) and incubated with 100 μL of 1% BSA in PBST for 1 hour at room temperature. Plates were then washed with PBST and incubated with 100 μL of anti-Ebola mAbs (13C6, 2G4, 4G7, KZ52) at concentrations following a 3-fold dilution scheme for 2 hours. Plates were washed again with PBST and 100 μL of secondary antibody, Rabbit anti-Human IgG at a 1:5000 dilution for 1 hour. Finally, plates were washed, and incubated with 100 μL of TMB Substrate (KPL) for 5 minutes and quenched with 100 μL of 1 N H_2_SO_4_. Plates were read using a Spectramax S5e plate reader at 450 nm.

### Sequence Conservation Analysis

To analyze Ebola virus protein sequence conservation, GP sequences were downloaded from the NCBI Virus Variation database (https://www.ncbi.nlm.nih.gov/genome/viruses/variation/). For the EBOV GP conservation analysis, analyzed sequences included human Zaire ebolavirus GP that were collected prior to August 2015, for a total of 955 unique protein sequences. For filovirus conservation analysis, representative protein sequences of filovirus coat glycoproteins were chosen: Zaire ebolavirus (genbank: AAB81004.1), Sudan ebolavirus (genbank: AGL73446.1), Reston ebolavirus (genbank: BAB69006.1), Tai Forest ebolavirus (genbank: ALT19763.1), Bundibugyo ebolavirus (genbank: AGL73474.1) and Marburg marburgvirus (genbank: ACT79243.1). Downloaded sequences were then aligned using MAFFT (www.ebi.ac.uk/Tools/msa/mafft/). Conservation percentages were obtained by importing the alignment into Jalview, and percentages were then mapped to the surface of EBOV GP (PDB: 3CSY) in PyMol for visualization. Coloring cutoffs were chosen from either 95–100% or 70–100% to highlight the most relevant surface trends.

### Inter-residue Epitope Network Analysis

The coordinates of EBOV GP trimer-antibody co-crystal structures (PDB IDs: 3S88, 3CSY, 3VE0) were uploaded into the PDBePISA server (http://www.ebi.ac.uk/msd-srv/prot_int/pistart.html) to determine residues located at the various intra-domain interfaces (between GP_1_ and GP_2_) and epitope-paratope interfaces. Further, the solvent accessibility of the residues was calculated using DSSP server (http://www.cmbi.ru.nl/dssp.html). For each residue the inter-residue interactions were calculated using a custom MATLAB script that incorporates putative hydrogen bonds (including water-bridged ones), disulfide bonds, pi-bonds, polar interactions, salt bridges, and Van der Waals interactions (non-hydrogen) occurring between pairs of residues within a threshold distance and has been described previously^20^. The threshold distances are as follows: H-bond donor-acceptor (oxygen and nitrogen), 4 Å; pi-bond (pair of phenyl rings from Tyr, Phe, Trp), 7 Å; disulfide bonds (pair of sulfur atoms), 2.5 Å; van der Waals interactions (pair of atoms other than oxygen, nitrogen, sulfur), 5 Å. These data were assembled into an array of eight atomic interaction matrices. A weighted sum of the eight atomic interaction matrices were then computed to produce a single matrix that accounts for the strength of atomic interaction between residue pairs, using weights derived from relative atomic interaction energies^20^. The weights are as follows: 20 (H-bond side chain-side chain), 10 (H-bond backbone-side chain), 5 (H-bond backbone-backbone), 10 (van der Waals side chain-side chain), 50 (van der Waals pi-pi), 5 (van der Waals backbone-side chain), 2.5 (van der Waals backbone-backbone) and 50 (disulfide). The inter-residue interaction network calculated in this fashion generates a matrix that describes all the contacts made by a given residue with spatial proximal neighboring residues in their environment. Each element *i, j* is the sum of the path scores of all paths between residues *i* and *j*. The degree of networking score for each residue was computed by summing across the rows of the matrix, which was meant to correspond to the extent of “networking” for each residue. This interactional relationship is represented using a two-dimensional network diagram. The degree of networking score was normalized with the maximum score for each protein so that the scores varied from 0 (absence of any network) to 1 (most networked). This entire process is depicted graphically in Supplementary Fig. S2. The network information was either mapped onto the surface of EBOV GP in PyMol, or a 2-D representation of the network (or a subset) was created using Cytoscape. Cocrystal Δ values for KZ52 bound to GP were calculated by running the residue interaction network analysis for both the KZ52-GP cocrystal structure and the unbound GP crystal structure, and subtracting the scores across the matched residues.

## Additional Information

**How to cite this article:** Quinlan, D. S. *et al*. An inter-residue network model to identify mutational-constrained regions on the Ebola coat glycoprotein. *Sci. Rep.* **7**, 45886; doi: 10.1038/srep45886 (2017).

**Publisher's note:** Springer Nature remains neutral with regard to jurisdictional claims in published maps and institutional affiliations.

## Supplementary Material

Supplementary Information

## Figures and Tables

**Table 1:** Summary of GP point mutations.

Mutation	GP Network score	KZ52 cocrystal Δ	EC50 (pM)			
			KZ52	2G4	4G7	13C6
2014 WT	n/a	n/a	118	105	46.1	49.1
V505A	0.036	0.102	314	*1560*	119	90.2
N506A	0.100	0.077	**169000**	**69300**	351	129
A507V	0.031	0.044	90.9	65.5	39.7	37.9
P509A	0.052	0.028	440	371	165	86.5
K510A	0.056	0.055	251	368	115	67.9
N514A	0.098	0.012	324	307	67.8	72.2
L547A	0.152	0.000	*3240*	*3310*	**20200**	140
H549A	0.108	0.076	303	136	48.8	46.1
N550A	0.097	0.057	**—**	**—**	*1950*	115
Q551A	0.073	0.043	276	373	60.6	65.1
D552A	0.063	0.125	**69900**	205	**—**	69.3
G553A	0.000	0.000	*4950*	**343000**	37.1	39.0
E564A	0.117	−0.005	582	653	144	100

Network score for a given residue is tabulated next to ELISA results for a mutation at that position. GP Network Score refers to the network score for that residue in EBOV GP. KZ52 cocrystal Δ refers to the increase or decrease in network score observed when the analysis was repeated for the KZ52-GP cocrystal structure (PDB: 3CSY). Binding data is colored as follows: 3-fold difference (underlined), 10-fold difference (shown in italics), >100-fold difference (shown in bold). All EC50 values are represented in picomolar and represent the average of three independent experiments. ‘—’ indicates that no fit was obtained.
