# Supplementary material for: An inter-residue network model to identify mutational-constrained regions on the Ebola coat glycoprotein
Source: Sci Rep. 2017 Apr 11;7:45886. doi: 10.1038/srep45886 (PMC5387726; doi:10.1038/srep45886)
Supplement: Supplementary Information [file srep45886-s1.pdf]

Supplemental Information

Title

An inter-residue network model to identify mutational-constrained regions on the Ebola coat glycoprotein

Authors

Devin S. Quinlan[1,2], Rahul Raman[1,2], Kannan Tharakaraman[2], Vidya Subramanian[2], Gabriella Del Hierro[2,3], Ram Sasisekharan[1,2;] *

Affiliations

1. MIT Department of Biological Engineering, 2. Koch Institute for Integrative Cancer Research at MIT, 3. MIT Department of Chemical Engineering. * corresponding author.

Contact Information

Email: rams@mit.edu

Phone:

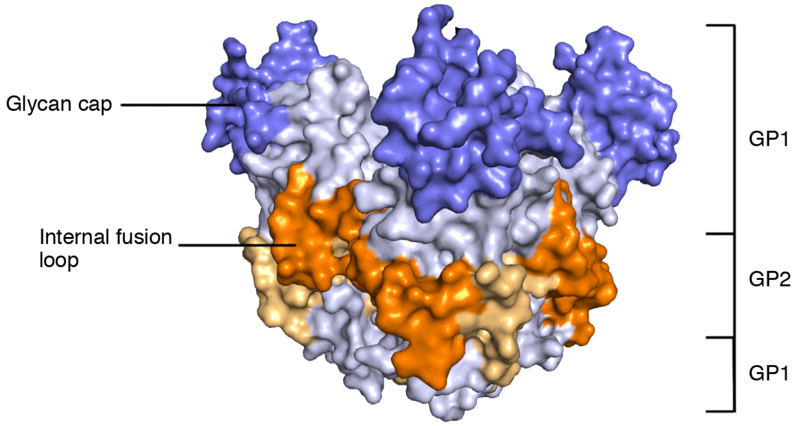


Supplementary Figure S1. Related to Figure 1. Regions of interest on Ebola GP. The crystal structure (PDB: 3CSY) is shown with the glycan cap and internal fusion loop highlighted in bright blue and bright orange, respectively. The GP1 and GP2 subunits are differentiated by blue and orange, respectively. A more detailed depiction can be found in the original characterization of this protein by Lee et al., 2008.[3]

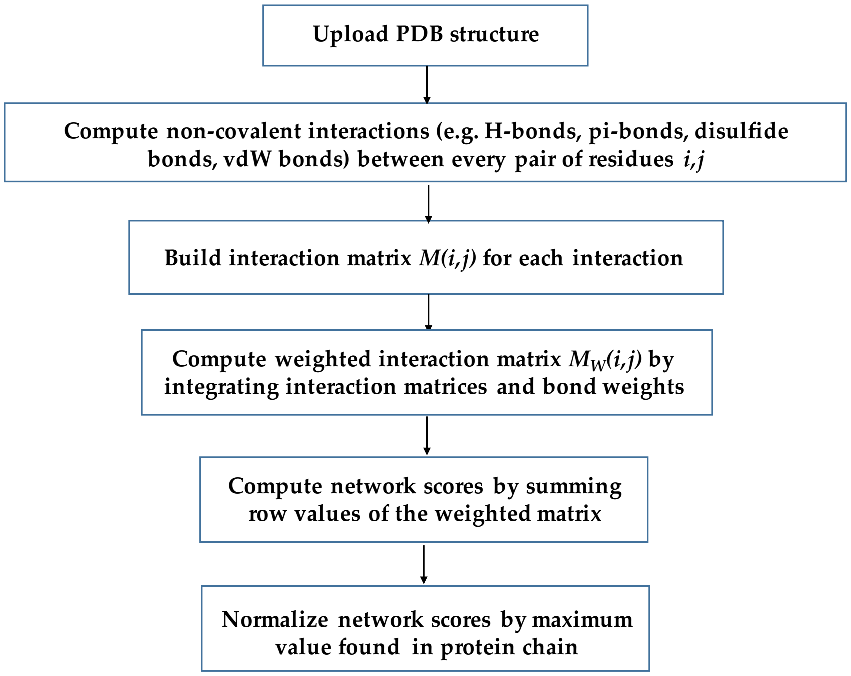


Supplementary Figure S2. Related to Figure 1. Flowchart depicting methodology for determining network scores for a residue within a protein crystal structure. A more detailed explanation is described in the Methods section.

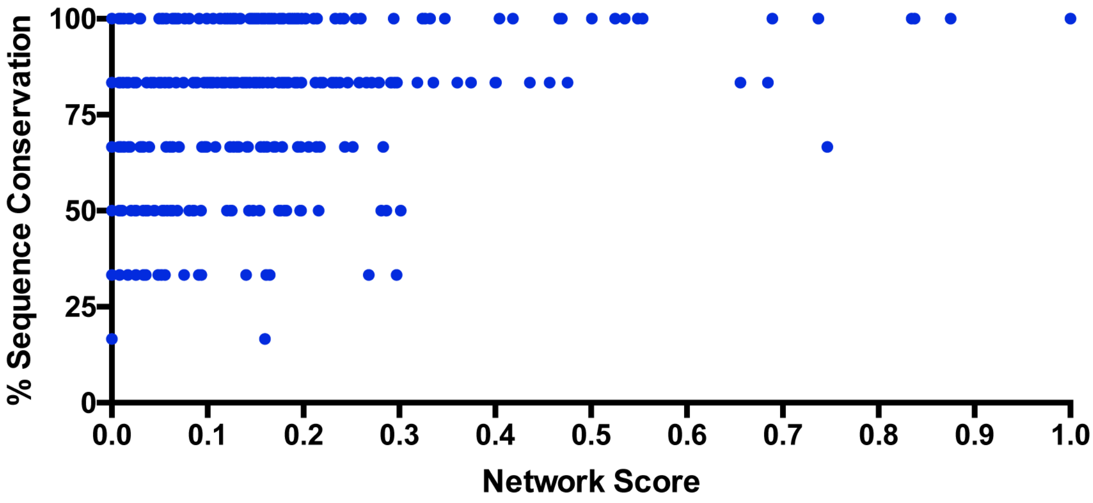


Supplementary Figure S3. Related to Figure 2. Sequence conservation across all filovirus glycoproteins vs. network score. Protein sequence conservation percentages are plotted against the normalized network score for that particular residue. There is a general trend that while low network-scoring residues may be either highly or poorly conserved, as network score increases, there are fewer residues with low observed sequence conservation. Beyond a 0.35 network score, there are no residues with under 50% sequence conservation within the *filoviridae* family.

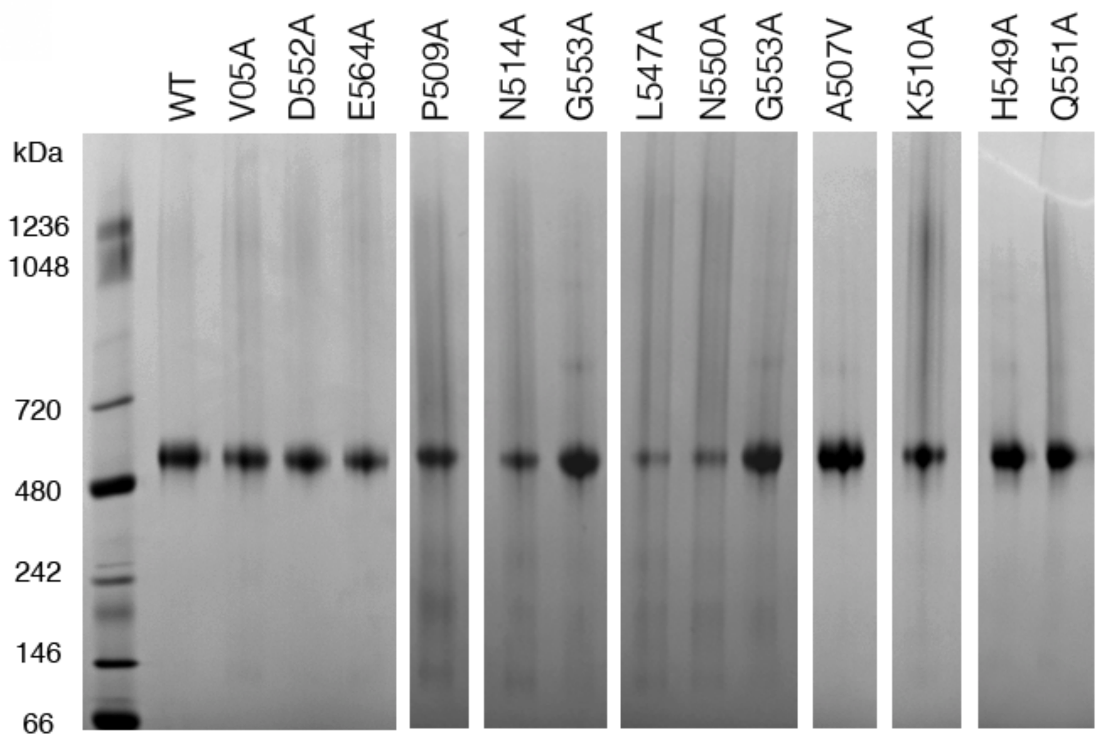


Supplementary Figure S4. Related to Figure 4 and Table 1. Native gel images of GPΔTMΔmuc and representative mutants. Lane 1 contains the NativeMark ladder and lanes 2-5 contain glycosylated, trimeric GP. Purified GPΔTMΔmuc can be seen as a single, trimeric species that runs at a consistent molecular weight for the WT and mutant species. The kDa value measured using the NativeMark ladder does not match up with the predicted molecular weight of the trimeric protein. This phenomenon has been observed in previous literature[7] and the correct monomeric molecular weight was validated using MALDI mass spectrometry to be ~340 kDa.

| **Virus** | **Mutation rate (nucleotide substitutions per site per year)** |
|---|---|
| Ebola | $1.25 \times 10^{-3}$ |
| Dengue | $4.55 \times 10^{-4}$ – $9.01 \times 10^{-4}$ |
| Influenza (H1N1) | $1.1 \times 10^{-3}$ – $3.2 \times 10^{-3}$ |

Supplementary Table S1. Related to Figure 1. Reported mutation rates (nucleotide substitutions per site per year) across several relevant RNA viruses (Carter and Sanford, 2012; Dunham and Holmes, 2007).

Site-directed mutagenesis / alanine scan

| *mAb* | *known hotspot* | Median network score | Median secondary network score | Median secondary network score (surface residues) |
|---|---|---|---|---|
| 1H3 | 275, 276, 279 | 0.010 | 0.041 | 0.070 |
| 13C6 | 270, 272 | 0.008 | 0.072 | 0.017 |
| 6D8 | 394, 395, 396, 397, 398, 400 | -- | -- | -- |
| 13F6 | 406, 409, 411, 412 | -- | -- | -- |
| 2G4 | 511, 550, 553, 556 | 0.112 | 0.158 | 0.156 |
| 4G7 | 511, 552, 556 | 0.118 | 0.141 | 0.132 |
| KZ52 | 511, 550, 552, 553, 556 | 0.118 | 0.141 | 0.129 |
| #3327 ** | 528 | 0.000 | 0.248 | 0.207 |

Crystral Structures

| *mAb* | *interface residues* | |
|---|---|---|
| KZ52 | 40, 41, 42, 43, 44, 502, 503, 504, 505, 506, 507, 508, 509, 510, 511, 513, 514, 549, 550, 551, 552, 553, 554, 556 | 0.130 |
| mAb100 | 31, 32, 34, 45, 46, 47, 49, 503, 505, 507, 508, 521, 522, 523, 524, 525, 526, 527, 544, 560, 563, 564, 565, 567, 568, 571 | 0.037 |
| mAb114 | 112, 114, 115, 116, 117, 118, 119, 120, 142, 143, 144, 145, 146, 147, 172, 221, 222, 223, 224, 227, 229, 231, 238, 239, 241, 269, 309 | 0.063 |

Supplementary Table S2. Related to Figure 3. Summary of network score information for known anti-Ebola antibodies binding to EBOV GP. Hotspot and interface residues as well as their associated network scores refer to the residues within EBOV GP (pdb: 3CSY). Hotspot residues for 1H3, 13C6, 6D8, 13F6, 2G4, 4G7 and KZ52 were described in Davidson et al., 2015. Hotspot residue for antibody #3327 was described in Reynard and Volchkov, 2015. Crystal structure information was taken from publically available crystal structures: KZ52 (pdb: 3CSY), mAb100 and mab114 (pdb: 5FHC). Table is colored based on network score. '—' indicates that network information could not be obtained, as the hotspot residues were not part of the GP crystal structure. ** Antibody #3327 secondary network score was determined using residues 527 and 529, since the known hotspot at glycine 528 does not make side-chain interactions.
